# Oral Microbiome and Edentulism During Pregnancy: 16S rRNA Gene Analysis of an Indigenous Community—A Pilot Study

**DOI:** 10.3390/microorganisms13091966

**Published:** 2025-08-22

**Authors:** Pablo Vásquez-Toasa, Juan C. Fernández-Cadena, Derly Andrade-Molina

**Affiliations:** 1 Omics Sciences Laboratory, Faculty of Health Sciences, Universidad Espíritu Santo, Samborondón 092301, Ecuador; pvasquezt@uees.edu.ec (P.V.-T.);; 2 Harvard Medical School, Brigham and Women’s Hospital, Boston, MA 02115, USA

**Keywords:** dentin, oral mucosa, edentulism, pregnancy, non pregnancy, Salasaca

## Abstract

Background: Edentulism, or toothlessness, is a significant public health issue with profound implications for physical and systemic health, especially during pregnancy, when hormonal and behavioral changes increase the risk of oral diseases. Indigenous populations are particularly vulnerable due to socioeconomic and cultural factors that limit access to dental care. Methods: This pilot study assessed the oral microbiota of nine women, both pregnant and non-pregnant, aged 18–35 from the Salasaca indigenous community in Ecuador, using 16S rRNA gene sequencing. Samples were collected from dentin, saliva, and oral mucosa, and analyzed for alpha and beta diversity levels, taxonomic composition, and ecological metrics using the DADA2 pipeline and a canonical correspondence analysis. Results: Pregnant participants exhibited significantly lower microbial diversity compared to non-pregnant individuals, with notable differences in species richness and community structure. Dominant phyla included Bacillota, Bacteroidota, and Pseudomonadota. *Prevotella* sp., *Neisseria* sp., and *Haemophilus* sp. were among the prevalent genera, with the canonical correspondence analysis highlighting associations between microbial profiles and variables such as gestational status, marital status, and BMI. Conclusion: The findings suggest that pregnancy influences the oral microbiota composition, potentially predisposing women to dysbiosis and dental pathology. This study highlights the need for targeted oral health strategies during pregnancy and serves as a foundation for larger studies in underserved indigenous populations.

## 1. Introduction

Edentulism, defined as the partial or complete loss of natural teeth, represents a complex public health issue with significant implications for individual well-being and social functioning [1]. Its etiology is multifactorial, often involving bacterial infections, genetic predispositions, dietary habits, and culturally rooted practices—particularly among indigenous populations [1]. Pregnant women represent a particularly vulnerable group, as physiological and hormonal changes during pregnancy increase the risk of periodontal disease [1]. Elevated levels of estrogen and progesterone can lead to gingival inflammation and heightened sensitivity, making the oral cavity more susceptible to infections and dental caries. Additionally, altered eating behaviors and common pregnancy symptoms—such as nausea and vomiting—can further compromise oral health during this critical stage.

The consequences of poor oral health during pregnancy extend beyond individual discomfort and may affect both maternal and fetal health. Studies have linked maternal periodontal disease to adverse pregnancy outcomes, including preterm birth and low birth weight. Addressing these disparities is, therefore, crucial not only for the mother’s well-being but also for optimizing birth outcomes and infant development.

Oral health disparities among Indigenous populations persist, and in some regions are even widening. Access to dental care in these communities is shaped by a complex interplay of historical trauma, biological vulnerability, sociocultural practices, political marginalization, and economic constraints [2]. Understanding oral health in Indigenous groups requires attention to two key dimensions: (i) Dietary patterns tied to subsistence economies, as many Indigenous communities follow nutritional practices influenced by trade, food availability, and tradition. Some rely on up to 50 different crops, with bananas and cassava being the most prevalent [3]. (ii) Oral hygiene behaviors, which depend on access to commercially available products such as toothbrushes, toothpaste, and dental floss—items often unaffordable for low-income populations.

The indigenous population’s low socioeconomic status significantly limits access to oral hygiene supplies, particularly among vulnerable groups such as pregnant women, who often cannot afford basic items such as toothbrushes, toothpaste, or dental floss [4]. Oral lesions are frequently observed during pregnancy, with studies reporting a high prevalence of dental alterations—most notably dental caries (99.38%) and erosions [5]. The mechanisms underlying tooth loss during pregnancy remain unclear; however, evidence suggests that it is less attributable to pregnancy itself and more likely due to disruptions in oral hygiene practices [6]. Gingivitis and gingival hypertrophy have been widely documented, and because such conditions are common in this period, some healthcare professionals perceive gum bleeding and enlargement as “normal” findings. Gingival inflammation has been reported, with prevalence rates ranging from 50% to 98.25% [6]. Periodontitis is also frequently observed in pregnant women, and several authors have noted increased tooth mobility during pregnancy.

The composition of oral microbial communities has been associated with periodontal disease during pregnancy. Notably, microbial shifts have been observed throughout gestation, with some studies reporting the displacement of commensal species such as Lautropia mirabilis and Prevotella melaninogenica in early pregnancy. The recurrence of P. melaninogenica has even been linked to premature birth [7]. These microbial changes are influenced by both nutritional status and oral health conditions during pregnancy [8]. Despite growing evidence linking pregnancy to changes in the oral microbiome and periodontal deterioration, there remains a significant knowledge gap regarding these phenomena in Indigenous pregnant women.

Indigenous communities, edentulism, and the COVID-19 pandemic share overlapping impacts and challenges, each representing public health concerns that disproportionately burden specific populations and amplify existing health disparities. Addressing these issues requires a comprehensive, multidimensional strategy that integrates medical, social, and cultural considerations while developing interventions tailored to the specific needs of each community. In particular, cultural customs and traditions within Indigenous groups may contribute to oral health deterioration [8].

In Ecuador’s Salasaca Indigenous communities, deep-rooted traditions are closely linked to festivals and culinary practices. The local diet primarily consists of corn-based foods, such as corn on the cob and roasted pork, often prepared over charcoal alongside yucca [8]. Dental evaluations in the region revealed suboptimal hygiene and dietary habits in both pregnant and non-pregnant women. Many pregnant women also exhibited multiparity, a factor associated with hormonal fluctuations that may predispose them to pregnancy-related gingivitis. Moreover, studies have documented elevated fluoride levels in local water sources, contributing to fluorosis and posing a risk for long-term enamel demineralization and joint problems across generations.

This exploratory investigation aimed to analyze the relationship between the oral bacterial microbiota of pregnant and non-pregnant women from an Indigenous population in Ecuador affected by edentulism. In the present investigation, the term “affected by edentulism” is comprehended within its broader epidemiological context, denoting the notable prevalence and historical context of partial tooth loss in the examined population, rather than suggesting that every participant was edentulous. The criteria for inclusion permitted women without any current missing teeth provided they possessed a documented history of dental extractions, facilitating a comparative analysis between individuals experiencing partial tooth loss (edentulous in at least one quadrant) and those preserving the majority or entirety of their dentition. This methodology sought to encompass the complete range of oral health conditions and to investigate how pregnancy-related microbial alterations might interact with various levels of tooth retention, acknowledging that the majority of pregnant women within the cohort retained most of their teeth [9,10].

Using next-generation sequencing (NGS) technologies and targeting the 16S ribosomal RNA (rRNA) gene, we achieved accurate taxonomic profiling of the oral microbiome. This culture-independent approach enabled a comprehensive view of the microbial communities implicated in edentulism within the context of pregnancy. Given the social and logistical challenges inherent in working with underserved Indigenous populations, the study was designed as a pilot, not to yield generalizable conclusions, but to establish a robust methodological framework and generate preliminary data. Furthermore, given the significant impact of the early-life oral microbiome on long-term health outcomes, a succinct examination is warranted. A contemporary systematic review indicates that a fundamental oral microbiome is established in neonates and exhibits relative stability in its species composition throughout early childhood (from birth to 18 years), albeit factors such as delivery method and feeding practices may affect its progression [11]. Incorporating this perspective emphasizes the necessity of investigating how pregnancy-related alterations in the maternal oral microbiome could potentially shape the initial microbial colonization in offspring—particularly in indigenous and marginalized communities [12].

These initial insights aim to inform future large-scale investigations targeting similar vulnerable communities.

## 2. Materials and Methods

### 2.1. Recruitment of Participants

Participants were voluntarily recruited by the Omics Sciences Laboratory at Universidad Espíritu Santo in collaboration with the Salasaca Health Center Type B, located in Tungurahua Province, Ecuador. The study included indigenous women, pregnant or non-pregnant, aged between 18 and 35 years. Information collected from each participant included general and dental surgical history, tobacco or alcohol use, number and average interval of previous births, frequency of dental visits, previous dental conditions, and types of dental hygiene tools used. Exclusion criteria were mestizo women, those with visible dental damage (e.g., cracked teeth, cavities, halitosis, gum disease), or dentin contaminated by infectious lesions.

Criteria were modified due to the high incidence of untreated oral diseases in the community. Participants with mild to moderate dental issues were included, provided there were no acute infections or extensive lesions affecting dentin sampling. This modification facilitated a more accurate representation of oral health in the target population while preserving the integrity of the microbiome analysis.

Clinical characteristics such as age, weight, height, and body mass index (BMI) were also recorded. The average BMI was 25.05, classifying participants as overweight according to the following categories: underweight (<18.5), normal (18.5–24.9), overweight (25–29.9), and obese (≥30).

### 2.2. Microbial Sampling Collection

Microbial samples were collected using a standardized, aseptic protocol. Dentin was obtained through curettage of the first and second molars using sterile curettes. This procedure followed the methodology recommended for high-throughput oral microbiome sequencing, where hard tissue sampling is prioritized after collecting saliva and mucosal swabs to minimize cross-contamination [1]. The dentin fragments were placed into sterile Petri dishes and immediately sealed.

Additionally, saliva, buccopharyngeal swabs, and oral mucosa samples (primarily from the inner cheek lining) were collected using sterile swabs. Each swab was placed into a 1.5 mL cryotube containing 150 μL of preservation buffer and stored at −80 °C until DNA extraction. All samples were collected under standardized and simultaneous conditions for both pregnant and non-pregnant women to ensure comparability across groups.

### 2.3. DNA Extraction and 16S rRNA Gene Sequencing

#### 2.3.1. DNA Extraction

Buccal and dentine DNA extraction was performed using the PureLink™ Genomic DNA Mini Kit (Thermo Fisher Scientific, Waltham, MA, USA), following the manufacturer’s protocol with modifications. Swab samples were initially placed in 1 mL of 1X PBS and stored at −80 °C until processing. For extraction, 200 μL of the sample was transferred to a 1.5 mL microtube containing 200 μL of a protoplast buffer (3.5 M sorbitol, 0.5 mg/mL lysozyme, 10 mM β-mercaptoethanol). Subsequently, 0.6 μM beads were added, and the sample was homogenized using a Bead Ruptor 4 (Omni International) at speed 5 for 180 s, repeated five times.

The mixture was then incubated at 37 °C for 1 h, followed by the addition of 180 μL of digestion buffer and 20 μL of fungal proteinase K (20 mg/mL). After vortexing, the samples were incubated at 65 °C for 45 min. RNase A (20 μL) was then added and incubated at room temperature for 10 min. Next, 200 μL of genomic lysis or binding buffer was added and incubated at 55 °C for 10 min. The mixture was again processed in the Bead Ruptor 4 (3 v, 180 s ×5), then centrifuged at 5000 rpm for 5 min. The supernatant was transferred to a clean microtube, mixed with an equal volume of ethanol, and passed through a PureLink Spin Column by centrifugation at 10,000× *g* for 1 min. This step was repeated as needed. DNA purification proceeded as described in the kit instructions, and the DNA quantity was measured using a Qubit™ 4 Fluorometer with the dsDNA BR Assay Kit (Thermo Fisher Scientific, Waltham, MA, USA).

#### 2.3.2. 16S rRNA Amplicon Sequencing

The V4 hypervariable region of the 16S rRNA gene was amplified by PCR using the forward primer 5′-TCGTCGGCAGCGTCAGATGTGTATAAGAGACAGCCTACGGGNGGCWGCAG-3′ and reverse primer 5′-GTCTCGTGGGCTCGGAGATGTGTATAAGAGACAGGACTACHVGGGTATCTAATCC-3′. Each reaction contained 1× reaction buffer, 2 mM MgCl_2_, 0.3 mM of each dNTP, 0.2 μM of each primer, 2.5 U of Kapa Taq DNA Polymerase (Kapa Biosystems, Wilmington, MA, USA), and 1–5 ng of template DNA in a final volume of 35 μL. The thermal cycling conditions included an initial denaturation at 94 °C for 3 min, followed by 28 cycles of denaturation at 94 °C for 30 s, annealing at 57 °C for 1 min, and extension at 72 °C for 1.5 min, ending with a final extension at 72 °C for 10 min.

Amplicons were verified by 2% agarose gel electrophoresis at 70 V. Triplicates of each sample were pooled and quantified via qPCR using the Library Quant Kit (Illumina, San Diego, CA, USA, Kapa Biosystems), then equimolarly pooled for sequencing. Library preparation was performed using a DNA PCR-Free Sample Preparation Kit (TruSeq^®^, Illumina, USA), with index codes added. Sequencing was carried out on the Illumina MiSeq platform using a 300-cycle kit. A total of 250 bp paired-end reads (PERs) were generated, demultiplexed using unique barcodes, and analyzed. The resulting sequences are available in the NCBI Sequence Read Archive (SRA) under accession number PRJNA1097549.

### 2.4. Bacterial 16S rRNA Gene Sequence Preprocessing and Analysis

The 16S rRNA gene sequences obtained from the MiSeq and HiSeq platforms were processed using the DADA2 pipeline (v1.28.0) in RStudio (v2023.06.2) [13]. Raw sequences were initially demultiplexed and assigned to each sample by matching barcode sequences via a custom data.frame script. After quality filtering, sequences with ambiguous bases, lengths outside the range of 200–300 bp, or homopolymers longer than eight nucleotides were excluded. The remaining high-quality reads were merged, and chimeric sequences were removed using the removeBimeraDenovo function [14]. Amplicon sequence variants (ASVs) were inferred with a minimum sequence identity cutoff of 97% using the furthest neighbor algorithm, and taxonomic classification was performed using the SILVA database (v138.2, released December 2025; https://www.arb-silva.de, accessed 1 April 2025) and the Human Oral Microbiome Database (HOMD, v15.2; http://www.homd.org, accessed 1 April 2025) [13,15]. To confirm the depth of sequencing, rarefaction curves were generated for each sample, and ASV abundance tables were normalized to the sample with the fewest reads. Visualizations such as species accumulation plots, rank abundance curves, and heatmaps were produced using ggplot (v3.4.2) in R (version 2.15.3).

Alpha diversity metrics, including Shannon and Simpson indices, were calculated using the summary single script in DADA2 [13]. Beta diversity between groups was computed using the Bray–Curtis dissimilarity algorithm. For statistical analyses, hierarchical clustering and SIMPROF (similarity profile analysis) methods were used to test for structural patterns without prior group definitions, implemented through the clustsig package (v1.2.3) [16,17]. To assess differences between predefined groups—such as pregnant vs. non-pregnant or edentulous vs. dentate women—the ANOSIM (analysis of similarities) test was applied using the vegan package (v2.6-4) in R [18]. All steps were performed within the phyloseq environment (v1.30.0), ensuring a reproducible and integrated microbial ecology workflow.

## 3. Results

### 3.1. Participant Characteristics and Clinical Overview

A pilot study was conducted at the Type B Health Center in the Salasaca community in September 2022. The study included nine female participants, aged 18 to 35 years—four pregnant (EM) and five non-pregnant (NEM)—all with a history of tooth loss and prior COVID-19 diagnosis. Most participants had completed secondary education and were in a marital union. Regarding lifestyle habits, 89% reported abstinence from alcohol, and none consumed psychoactive substances.

Dental and sequencing data were obtained from these nine participants, each contributing one or two biological samples, for a total of 14 specimens. Each participant provided at least one saliva sample, and in five cases, an additional dentin sample was collected (indicated by the suffix “D” in the sample ID). The IDs also reflect pregnancy status, whereby EM denotes a pregnant participant and NEM a non-pregnant one. Not all participants contributed both types of samples.

Clinical dental evaluations revealed that all participants presented with dental caries, along with additional conditions such as gingivitis or mild gum inflammation, dental calculus, periodontitis, pericoronitis, and pulpitis. The numbers of missing teeth ranged from 0 to 4, with a median of 1 missing tooth per participant. Reported tooth brushing frequency rates varied from once a week to three times daily, with most participants brushing at least twice per day. Dentin samples lacked associated clinical metadata but were included in sequencing and diversity analyses.

Details for each participant and sample are presented in Table 1. Additional sociodemographic and oral hygiene behavior data—including age, education level, occupation, BMI, frequency of dental visits, and oral hygiene tools used—are available in Table A1 and Table A2.

### 3.2. Microbial Diversity in Pregnant and Non-Pregnant Patients

After removing undesired data, preprocessing, and taxonomic classification, the dataset decreased from 3,356,949 to 1,226,953 sequences, resulting in a total of 1396 amplicon sequence variants (ASVs). Rarefaction curves demonstrated satisfactory sequencing depth, suggesting adequate coverage of microbial diversity in both pregnant (EM) and non-pregnant (NEM) participants (Table 1).

**Figure 1:**
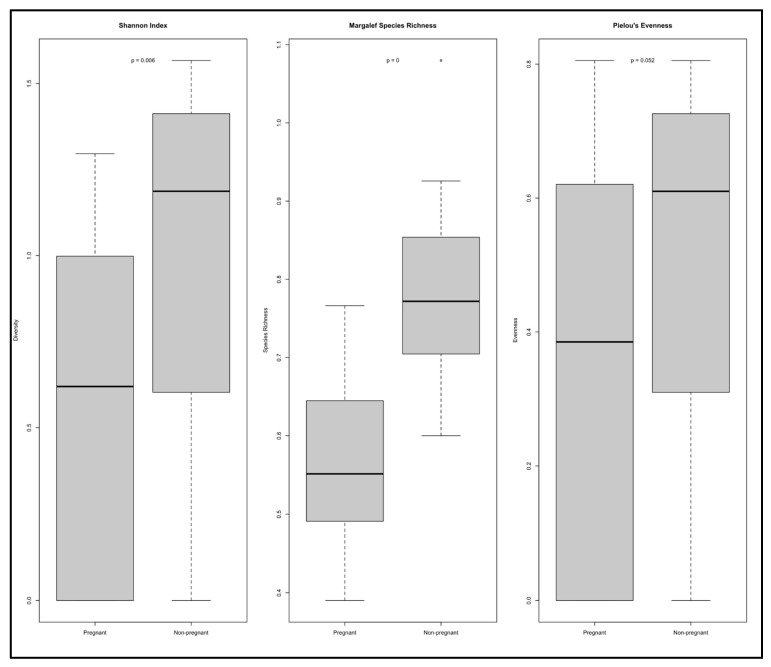
The Shannon diversity indexes (alpha diversity) of pregnant (EM, *n* = 4) and non-pregnant (NEM, *n* = 5) participants were compared using an unpaired Student’s *t* test, revealing a lower microbial diversity for the saliva from the edentulous subjects. Margalef’s index of species richness was also significantly different. Pielou’s index of evenness showed no difference.

Alpha diversity was assessed using three ecological indices: Shannon, Margalef, and Pielou (Figure 1). The Shannon diversity index revealed significantly lower microbial diversity in the EM group compared to the NEM group (*p* = 0.006). Similarly, the Margalef index showed a significant reduction in species richness among pregnant participants (*p* = 0). In contrast, Pielou’s evenness index did not reveal a statistically significant difference between the two groups (*p* = 0.092).

Across the three indices, non-pregnant participants exhibited consistently higher values, indicating greater diversity and richness. Summary metrics, read counts, and ASV numbers for each sample are detailed in Table 1.

### 3.3. Bacterial Community Composition in Pregnant and Non-Pregnant Individuals

**Figure 2:**
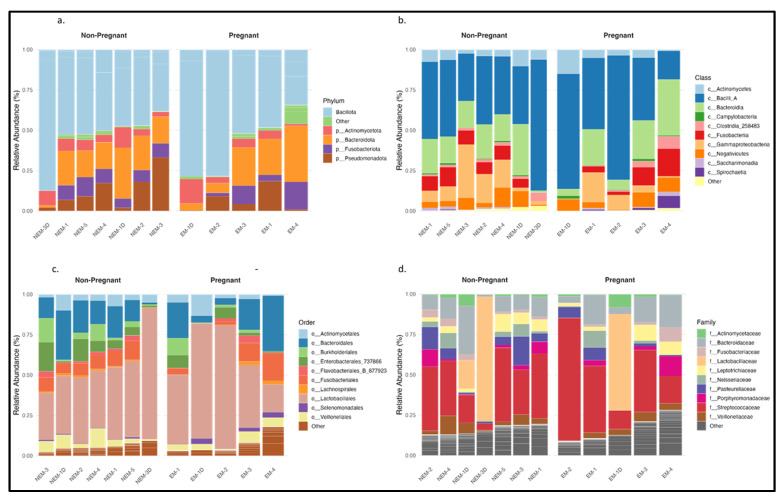
Relative abundance levels of the top 1396 genes between pregnant and non-pregnant subjects from dental and oral samples: (**a**) phylum; (**b**) class; (**c**) order; (**d**) family.

The taxonomic analysis of the 1396 ASVs identified in saliva and dentin samples revealed differences in microbial composition between pregnant (EM) and non-pregnant (NEM) participants. Figure 2 shows the relative abundance of bacterial taxa across four taxonomic levels. At the phylum level (Figure 2a), the dominant groups were Bacillota, Bacteroidota, Pseudomonadota, Actinomycetota, and Fusobacteriota, with Bacillota and Bacteroidota representing the majority. Pseudomonadota showed greater abundance in samples from pregnant individuals. At the class level (Figure 2b), Bacilli, Bacteroidia, Clostridia, Gammaproteobacteria, and Actinobacteria were most common. Bacilli appeared more frequently in the EM group. At the order level (Figure 2c), Lactobacillales, Clostridiales, Bacteroidales, and Actinomycetales were consistently present in both groups, with Lactobacillales showing increased abundance among EM samples. At the family level (Figure 2d), notable differences were observed in Streptococcaceae, Lactobacillaceae, Fusobacteriaceae, Actinomycetaceae, and Pasteurellaceae, the latter being more represented in the NEM group. These patterns align with previously reported associations between pregnancy and increased levels of acidogenic or proinflammatory taxa [7,14,19,20].

**Figure 3:**
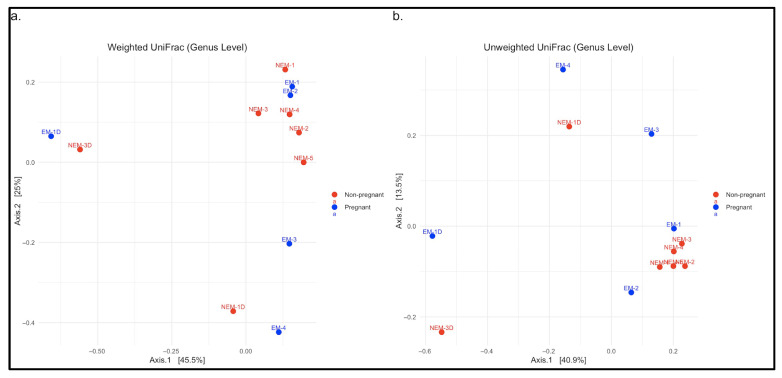
Diversity comparisons in saliva samples from pregnant (EM) and non-pregnant (NEM) participants at the genus level using UniFrac distance matrices. (**a**) Weighted UniFrac distance matrix, based on relative abundances of genera. (**b**) Unweighted UniFrac distance matrix, based on presence/absence of genera.

To further evaluate group differences, beta diversity was assessed using genus-level UniFrac distances. Figure 3a displays a principal coordinate analysis (PCoA) based on the weighted UniFrac method, indicating partial separation between EM and NEM samples and greater dispersion among EM samples, suggesting higher interindividual variability. In Figure 3b, the unweighted UniFrac plot shows similar clustering patterns, albeit less distinctly. These analyses confirm compositional shifts in microbial communities associated with pregnancy status. In total, nine phyla were identified across all samples, and taxonomic shifts were observed across all levels analyzed, particularly in taxa associated with acid production and inflammation [20,21].

### 3.4. Most Representative ASVs Between Pregnant and Non-Pregnant Samples

**Figure 4:**
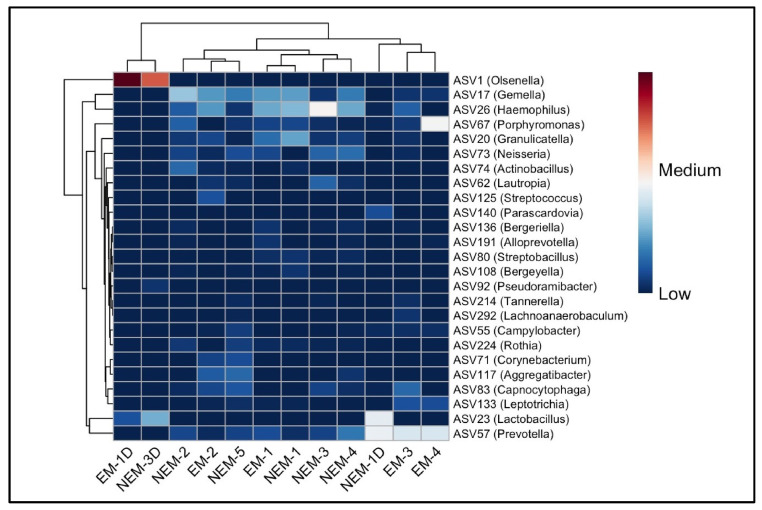
Heat map of the 25 most abundant genera in each sample to show the distribution of abundant ASVs. The distribution of ASVs is represented by the color intensity of each grid (blue, low abundance; red, high abundance).

Following the observed taxonomic differences between groups at the phylum, class, order, and family levels (Figure 2), we examined the specific amplicon sequence variants (ASVs) that were most abundant across samples. A total of 52 major ASVs were identified, and the top 25 are visualized in a heatmap (Figure 4). ASV1, classified as Olsenella, showed markedly higher abundance in dentin samples, especially EM-1D and NEM-3D, suggesting a distinct microbial composition in the dentin microenvironment. Other prominent ASVs included Gemella (ASV17), Haemophilus (ASV26), Porphyromonas (ASV67), Granulicatella (ASV26), and Streptococcus (ASV125), distributed among both groups, but with variable abundance levels.

Notably, EM-4 exhibited a unique microbial profile with elevated levels of Porphyromonas, observed exclusively in that sample. ASVs such as Prevotella, Lactobacillus, Neisseria, and Rothia were also present, often differing in prevalence between dentin and saliva. Shared ASVs across both pregnant (EM) and non-pregnant (NEM) participants—such as Gemella and Haemophilus—were mainly detected in saliva. In contrast, dentin samples consistently showed increased representation of acidogenic or proteolytic genera including Olsenella and Lactobacillus. These findings illustrate site-specific microbial patterns within the oral cavity and emphasize the distinct composition of the dental versus mucosal microbiome in both physiological conditions.

## 4. Discussion

After rigorous filtering and taxonomic assignment, a total of 1,226,953 high-quality sequences were retained across all samples, resulting in 1396 amplicon sequence variants (ASVs). Rarefaction curves confirmed sufficient sequencing depth, suggesting that the microbial diversity of the oral cavity was effectively captured in both cohorts. The alpha diversity analysis revealed significantly lower microbial richness and evenness in pregnant (EM) individuals compared to non-pregnant (NEM) participants, as measured by Shannon, Margalef, and Pielou indices. These findings indicate a notable ecological shift in the oral microbiome associated with pregnancy, suggesting the presence of fewer and more unevenly distributed microbial taxa in the EM group. Such reductions in diversity have been linked in previous studies to dysbiosis and a greater risk of oral pathologies [1,2].

The NEM group not only exhibited higher mean diversity values but also greater interindividual variability, as reflected in wider interquartile ranges. This diversity may represent a more balanced and resilient microbial ecosystem, which is typically associated with oral health and resistance to pathogenic colonization [3]. In contrast, the EM cohort’s lower diversity and skewed species distribution may reflect a microbial imbalance, potentially driven by pregnancy-related hormonal, immunological, and metabolic changes. Notably, the Pielou index revealed a lower evenness in EM samples, suggesting that specific taxa may dominate the oral environment during pregnancy. Such dominance could contribute to increased vulnerability to dental caries or periodontal inflammation, as previously suggested in the literature [4,5].

In addition to the compositional shifts in the oral microbiota, the observed reduction in microbial diversity during pregnancy may be driven by a complex interplay of hormonal and metabolic changes. Elevated levels of estrogen and progesterone during gestation are known to increase vascular permeability and gingival inflammation, creating a favorable environment for microbial dysbiosis [22]. These hormonal fluctuations have been shown to promote the proliferation of acidogenic and proinflammatory taxa, including Porphyromonas gingivalis and Tannerella forsythia, both of which are strongly associated with periodontal disease and adverse obstetric outcomes such as preterm birth and preeclampsia [23].

Immunologically, pregnancy is characterized by a state of selective immune tolerance that permits fetal development while simultaneously altering host defense mechanisms. This immunological modulation may impair the host’s ability to regulate microbial overgrowth, thereby increasing susceptibility to opportunistic infections in the oral cavity [24]. The overrepresentation of Streptococcus mutans, Lactobacillus spp., and Olsenella spp. in pregnant women, as observed in this study, may be linked to this immunosuppressed state. Moreover, oral dysbiosis has been implicated in triggering systemic inflammatory responses capable of crossing the placental barrier and affecting fetal development [25,26].

These findings bear important clinical implications. The enrichment of taxa associated with caries and periodontal disease in the pregnant cohort underscores the need for targeted oral health interventions during pregnancy [27]. Extensive research suggests a correlation between maternal periodontitis and negative pregnancy outcomes, especially preterm birth and low birth weight, with mechanisms involving pro-inflammatory cytokines and bacterial translocation [7,28], although the relationship is contested due to variability in study methodologies and definitions of periodontitis severity, while our findings, despite a limited sample size, support the hypothesis that microbial shifts during pregnancy may elevate this risk, indicating a need for further longitudinal studies in similar indigenous cohorts [9].

Previous studies have established strong associations between maternal periodontal status and negative pregnancy outcomes, such as low birth weight and gestational hypertension [7,28]. The detection of Olsenella in dentin samples also supports its candidacy as a potential microbial biomarker for gestational dysbiosis, a concept that remains underexplored in maternal oral health research.

From a public health standpoint, incorporating oral microbiome monitoring into prenatal care programs could facilitate the early detection of microbial imbalances and enable timely intervention. Non-invasive sampling methods such as saliva collection for ASV profiling may be particularly useful in low-resource settings or rural populations, where access to dental care is limited [25]. Furthermore, disseminating these results to healthcare professionals and the surrounding community via straightforward, visually supported reports and preventive protocols may augment the relevance of the findings within both clinical and public health frameworks [29]. Concise, layman-friendly overviews of microbial risk determinants, coupled with actionable guidance on oral hygiene practices during gestation, would facilitate the more effective incorporation of this information into standard medical and dental assessments [30].

Such approaches can help bridge health disparities and offer population-specific insights into oral disease prevention strategies.

Finally, this study highlights the importance of adopting a One Health perspective when examining maternal microbiomes. Environmental, nutritional, and socio-cultural factors intersect with biological processes to shape microbial communities during pregnancy. The oral cavity represents a critical interface where systemic and microbial dynamics converge, with potential downstream effects on neonatal health. Future longitudinal studies integrating microbiome data with psychosocial and dietary assessments are needed to unravel the multifactorial influences on maternal and child health outcomes [31].

## 5. Conclusions

This exploratory study provides evidence that pregnancy alters the oral microbiome of women from an indigenous community, resulting in reduced microbial diversity, increased abundance of pro-inflammatory genera, and distinct structural reorganization of the oral bacterial community. These findings support the hypothesis that pregnancy may induce a state of oral dysbiosis, potentially increasing susceptibility to oral infections such as dental caries and periodontitis, and possibly influencing adverse pregnancy outcomes, including preterm birth or low neonatal birth weight.

Furthermore, the differential presence of specific taxa across dentin and mucosal surfaces highlights the importance of considering the anatomical origin of biological samples in microbiome research. The frequent detection of Bacteroides in overweight individuals and in those with excessive gestational weight gain suggests a possible link between oral microbiota and host metabolic status. Additionally, the presence of Olsenella and Prevotella may serve as microbial biomarkers associated with increased oral health risk during pregnancy.

Limitations of this study include the small sample size and its cross-sectional design, which limit the statistical power and prevent the establishment of temporal or causal relationships. For a pilot study, the limited cohort size (*n* = 9) is a critical constraint, as it reduces the ability to detect subtle microbial differences and increases the risk of type II errors. Nevertheless, this design fulfills its primary objectives of testing the feasibility and refining sampling protocols, as well as generating preliminary data to guide larger, hypothesis-driven studies.

The lack of clinical metadata for certain samples, particularly dentin, further restricted our interpretation. These factors necessitate cautious generalization of the findings.

Future research should incorporate longitudinal designs with larger, diverse cohorts and integrate clinical and behavioral data. In particular, studies should prioritize vulnerable populations with limited access to dental care. The development of personalized, microbiome-informed preventive strategies could help mitigate oral health disparities and improve maternal and neonatal outcomes in underrepresented communities.

## Figures and Tables

**Table 1:** Oral health and sequencing data from study participants.

ID	Reads (Raw)	Reads (Filtered)	Amplicon Sequence Variants (ASVs)	Missing Teeth	Dentist’s Diagnosis	Frequency of Tooth Brushing
EM-1	12,181	11,932	191	2	Cavities, periodontitis or inflammation and heavy gum bleeding, presence of dental calculus	3 times a day
EM-1D	31,779	31,756	72	-	-	-
NEM-1	14,168	14,101	123	2	Cavities, presence of dental calculus	Once a day
NEM-1D	8968	8896	87	-	-	-
NEM-2	15,579	15,361	277	4	Cavities, -presence of dental calculus	3 times a day
NEM-3	9701	9611	124	2	Cavities, pulpitis	Once a day
NEM-3D	32,139	32,125	110	-	-	-
NEM-4	13,541	13,417	170	0	Cavities, gingivitis or mild gum inflammation and bleeding, pericoronitis	Once a week
EM-2	9893	9704	165	1	Cavities, gingivitis or mild gum inflammation and bleeding, -presence of dental calculus	2 times a day
EM-3	9562	9485	117	0	Cavities, gingivitis or mild gum inflammation and bleeding, presence of dental calculus	2 times a day
NEM-5	12,376	12,296	135	0	Cavities, pericoronitis	2 times a day
EM-4	7242	7185	76	0	Cavities, gingivitis or mild gum inflammation and bleeding	2 times a day

**Figure A1:**
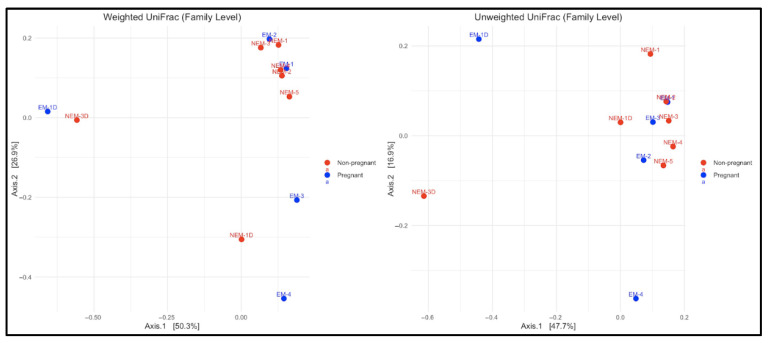
The principal coordinate analysis (PCoA) based on weighted (**left**) and unweighted (**right**) UniFrac distances at the family level. Each point represents an oral sample, coded by gestational status (blue for pregnant (**pregnant**) and red for non-pregnant (**non-pregnant**)). The separations between groups reflect differences in microbial composition. The wider scatter in pregnant women suggests greater variability between individuals.

## Data Availability

The original data presented in the study are openly available in in GenBank/EMBL/DDBJ under the BioProject accession number PRJNA1097549 (https://www.ncbi.nlm.nih.gov/bioproject/?term=PRJNA1097549, accessed on 14 July 2025).

## Abbreviations

EM	Pregnant
NEM	Non-pregnant
BMI	Body Mass Index
ASV	Amplicon Variant Sequence
CCA	Canonical Correspondence Analysis

## Appendix B

**Table A1:** Oral hygiene habits reported by study participants.

ID	How Long Ago Did You Brush Your Teeth?	How Often Do You Brush Your Teeth?	How Often Do You Visit the Dentist?	Oral Hygiene Tools Used in Each Brushing
EM-1	More than 6 h ago	3 times a day	Every 6 months	Toothbrush
NEM-1	3 h ago	Once a day	Every 6 months	Toothbrush
NEM-2	More than 6 h ago	3 times a day	Every 6 months	Toothbrush
NEM-3	More than 6 h ago	Once a day	Every 6 months	Toothbrush
NEM-4	3 h ago	Once a week	Every 6 months	Toothbrush
EM-2	3 h ago	2 times a day	Every 6 months	Toothbrush
EM-3	3 h ago	2 times a day	Once a year or more	Toothbrush
NEM-5	3 h ago	2 times a day	Once a year or more	Toothbrush
EM-4	3 h ago	2 times a day	Every 6 months	Toothbrush

**Table A2:** Sociodemographic, clinical, and dental characteristics of the study participants.

ID	Age	Occupation	Education Level	Marital Status	Consumes Alcohol	Number of Children	BMI	Location of Missing Teeth [2]
EM-1	18	Housewife	High School	Married	No	0	24.01	Quadrant #1: 12, 13 missing
NEM-1	25	Housewife	High School	Married	No	0	26.16	Quadrant #3: 34, 35 missing
NEM-2	28	Housewife	High School	Married	No	3	26	Quadrant #1: 18, 17, 14; Quadrant #2: 26 missing
NEM-3	22	Student	University	Single	Yes	0	23.68	Quadrant #2: 24, 25 missing
NEM-4	30	Housewife	Primary School	Married	No	2	27.2	0
EM-2	30	Housewife	High School	Married	No	1	29.4	Quadrant #4: 47 missing
EM-3	30	Housewife	High School	Married	No	1	24.3	0
NEM-5	24	Hairdresser	High School	Married	No	1	19.6	0
EM-4	18	Housewife	Primary School	Married	No	0	24.86	0
